# Open Access Publishing

**DOI:** 10.3201/eid1007.040122

**Published:** 2004-07

**Authors:** Jocelyn A. Rankin, Sandra G. Franklin

**Affiliations:** * Centers for Disease Control and Prevention, Atlanta, Georgia, USA;; † Emory University, Atlanta, Georgia, USA

**Keywords:** conference summary, open access publishing

An Open Access Publishing Conference was convened in Atlanta, Georgia, on January 7, 2004, by the libraries of the Centers for Disease Control and Prevention (CDC) and Emory University. Open Access is an emerging publishing model for peer-reviewed scientific research in which authors and their publishers grant free access to their work as long as the authors are acknowledged and the publisher ensures that the work is made freely available in a digital archive (*1*). The conference brought together key stakeholders including scientists, researchers, publishers, and librarians and included approximately 240 participants with 80 offsite registrants connecting through the simultaneous Web cast.

The keynote address, "The Coming Revolution in the Publication of Scientific Papers," delivered by Harold Varmus, emphasized that 1) in today's Internet era, the traditional Gutenberg print publishing model is outdated; 2) electronic publishing has the advantages of lower costs, global distribution, content that can be linked to datasets, improved archiving, and full-text searching; and 3) rigorous peer review is possible in electronic and Open Access formats. Open Access publishing challenges include engaging professional societies in this approach, building sustainable business plans, and changing academic culture so that published works are evaluated for content rather than for the journal label. Open Access publishing is typically financed by author fees along with a combination of philanthropic and advertising support. Examples are the Public Library of Science, Journal of Clinical Investigation, and BioMed Central journals. Recent milestones include the Bethesda Open Access Principles meeting (*1*), the Wellcome Trust endorsement of Open Access, and support from the Howard Hughes Medical Foundation and a number of leading European scientific societies.

A panel of speakers gave stakeholders' perspectives. Sheldon Kotzin reviewed the National Library of Medicine's (NLM) priorities regarding access to, and permanent retention of, the world's biomedical literature. Reflecting growing concerns about high costs of scientific publications, the U.S. Congress recently directed the NLM to report on the impact of rising journal subscription prices relative to access to medical research information and to identify remedies to ensure that taxpayer-funded research remains in the public domain. NLM's Open Access initiative is PubMed Central, a digital archive of freely available life sciences journals. After a slow start, the PubMed Central repository includes 137 journal titles. PubMed Central expects publishers to deposit full contents of each journal issue soon after publication. Supplementary data files are also encouraged. The recent addition of a single article from a journal that is not participating in PubMed Central is broadening the definition of this archive. Another Open Access approach was described by John Nickerson, editor of Emory University's Molecular Vision, which has been freely available on the Internet since its first issue in October 1995. A low-cost operation, Molecular Vision is a refereed open access journal that has achieved scientific recognition in its field.

Publishing trends affecting libraries were discussed by Linda Watson, University of Virginia Health Sciences Library, and included: 1) journal subscription price increases outpacing library budgets, 2) publishers' bundling of journal subscriptions into large contracts often not well matched with institutional research interests, 3) consolidations in the publishing industry, 4) restrictive licensing terms overriding copyright and fair use practices, 5) long-term archival access to electronic content, and 6) selective deletions of published articles from databases and e-publications. Presenting a scientist's perspective, CDC's Marta Gwinn noted that the scientific community's overarching responsibility is to ensure that research is conducted with integrity and quality and that access to it is fair, maximizes value to users, and protects the public investment and interests.

The open access conference generated discussion about the scientific research dissemination process and the need to strengthen the connections between evidence-based research and healthcare action. With high quality, peer-reviewed scientific research becoming freely available on the Internet, possibilities for more rapid advances in scientific knowledge and ultimately improved public health are important. Collaboration between government and academia is necessary to make progress toward open access to scientific research.

This conference was supported in part by the National Networks of Libraries of Medicine, Southeastern Atlantic Region. Conference presentations are available from: http://ada.healthsci.emory.edu/openaccess
